# Modeling and analysis of forward and inverse kinematics for a flexible Stewart platform

**DOI:** 10.1371/journal.pone.0353154

**Published:** 2026-07-06

**Authors:** Liyun Su, Linke Hou, Jiaodi Liu

**Affiliations:** 1 Education Department of Guangxi Zhuang Autonomous Region, Key Laboratory of Advanced Manufacturing and Automation Technology (Guilin University of Technology), Guilin University of Technology, Guilin, Guangxi, China; 2 College of Mechanical and Control Engineering, Guilin University of Technology, Guilin, Guangxi, China; Indian Institute of Technology Patna, INDIA

## Abstract

Stewart platforms are widely used in flight simulators, precision machining, and other fields due to their advantages in high precision, high dynamic response, and full six-degree-of-freedom spatial motion. However, the positioning accuracy of traditional rigid Stewart platforms is difficult to further improve due to limitations such as the structure of telescopic rods and insufficient kinematic solution accuracy. To address this technical challenge, this study proposes a flexible Stewart platform and conducts modeling and analysis on its forward and inverse kinematic solutions. First, by introducing piezoelectric ceramics to calculate the displacement loss caused by telescopic rods overcoming the inertia of the moving platform and load, a precise mathematical model for inverse kinematics is established based on geometric analysis and kinematic theory. Second, aiming at the problems of low efficiency and low accuracy in solving forward kinematics using the Newton-Raphson method and traditional BP neural networks, an improved BP neural network method based on the Levenberg-Marquardt (L-M) algorithm is innovatively proposed. By constructing a multi-layer feedforward neural network model and using inverse kinematic formulas to generate training datasets, a nonlinear mapping from rod lengths to platform pose is achieved, effectively avoiding the complexity of traditional calculation processes. Finally, MATLAB simulation results show that regarding inverse kinematics, the calculated displacement range of piezoelectric ceramics covers 27.9 nm to 47.4 nm. In terms of forward kinematics, the relative error of pose prediction using the proposed improved algorithm is controlled within 0.5% across the entire domain, with absolute errors in heatmaps controlled around 0.02 mm. The forward and inverse kinematic solution methods proposed in this paper for high-precision positioning flexible Stewart platforms are significantly superior to traditional methods in terms of friction displacement compensation range and pose prediction accuracy. This work not only provides an innovative solution for high-precision positioning technology but also lays an important theoretical foundation for applications in industrial robotics and precision measurement.

## Introduction

As a classic parallel mechanism, the Stewart platform has been widely applied in motion simulation, precision manufacturing, aerospace docking, and medical equipment since its proposal by D. Stewart in 1965 [1]. This widespread adoption is driven by its core advantages, including a high stiffness-to-mass ratio, strong load-bearing capacity, fast dynamic response, and excellent positioning accuracy [2]. However, as modern industry demands ever-increasing positioning precision, traditional rigid Stewart platforms face significant challenges. Their structural characteristics limit the solving process of both forward and inverse kinematics, making it difficult to meet the requirements for precise positioning.

Compliant mechanisms, characterized by friction-free operation, zero backlash, wear-free performance, and monolithic manufacturing, have found extensive applications in precision positioning, micro-nano manipulation, and biomedical engineering. The static and dynamic modeling of these mechanisms serves as the core foundation for performance analysis and optimization design. Currently, this field has established a relatively comprehensive theoretical system. Ling et al.[3] systematically reviewed the fundamental concepts, applicability, and inherent limitations of mainstream modeling methods—such as the Pseudo-Rigid-Body Model (PRBM), Compliance Matrix Method (CMM), Beam Constraint Model (BCM), and Finite Element Method (FEM)—under both small and large deformation conditions. The classic monographs by Howell et al.[4] and Lobontiu [5] laid the theoretical cornerstone for the design and modeling of compliant mechanisms. Meanwhile, Yong et al.[6], Wang et al.[7], and Awtar et al.[8] provided comprehensive summaries on specialized topics, including the precision modeling of flexure hinges, the design of constant-force mechanisms, and constraint design for parallel flexible systems. While existing research on small-deformation linear modeling is relatively mature, challenges remain in balancing computational efficiency and accuracy regarding the displacement loss of Stewart platforms during positioning, necessitating further in-depth investigation.

Precise positioning of the Stewart platform relies heavily on its inverse kinematics. By solving the inverse kinematics, the target extension of each driving leg can be determined, providing the theoretical basis for achieving the desired pose of the moving platform. However, traditional theoretical derivations for inverse kinematics typically rely on ideal geometric relationships, often overlooking various nonlinear factors present in actual systems. In particular, during the movement of the platform, the telescopic legs must overcome the gravity of the moving platform, which prevents it from reaching the ideal position and significantly affects the system’s actual output [9–11]. If this is not taken into account, a deviation will exist between the theoretical rod length and the actual required rod length. These uncompensated errors accumulate across the platform’s six legs, ultimately compromising the comprehensive positioning accuracy of the moving platform.

To achieve high-precision positioning of the moving platform, accurately solving the forward kinematics of the Stewart platform to determine its actual pose is crucial. However, traditional numerical methods (such as the Newton-Raphson method) exhibit significant limitations in solving this problem. They are highly dependent on initial values, and minor fluctuations in these values can easily cause the iteration process to fall into a non-singular matrix trap [12]. In contrast, traditional Back Propagation (BP) neural network methods do not rely on initial guesses. They can directly estimate the pose of the moving platform from the driving rod lengths through nonlinear mapping, thereby improving the dependence on initial values to some extent [13]. However, these methods also have their own limitations, including the need for large sample datasets during the training phase, time-consuming learning processes, and issues such as slow convergence speeds and large prediction errors during actual prediction. Although the aforementioned research methods can effectively solve the forward kinematics problem, they still present certain limitations in practical applications.

To effectively address the issues existing in the forward and inverse kinematics of traditional rigid Stewart platforms, this paper proposes a novel High-Precision Flexible Stewart Platform (FSP). This platform integrates piezoelectric ceramic patches with telescopic rods. Regarding inverse kinematics, the physical quantity generated by the moving platform overcoming the gravity of the platform and its load during motion is described as the elastic deformation of the piezoelectric ceramics. Subsequently, by calculating this deformation, compensation is performed for the displacement loss caused during movement [14,15]. In terms of forward kinematics, this paper innovatively proposes a pose parameter solution method for the moving platform based on an improved BP neural network using the Levenberg-Marquardt (L-M) algorithm. This approach aims to resolve the highly nonlinear relationship between the telescopic rod lengths and the platform pose, providing a more reliable solution for the forward kinematics calculation of the FSP. This research not only provides new insights for the design and optimization of high-precision Stewart platforms but also lays a foundation for the development of precision control theory for parallel mechanisms through its proposed displacement compensation mechanism and forward kinematics solution method, holding significant application value in fields such as precision positioning and manufacturing.

## Analysis of inverse kinematics

### FSP modeling

**Fig 1:**
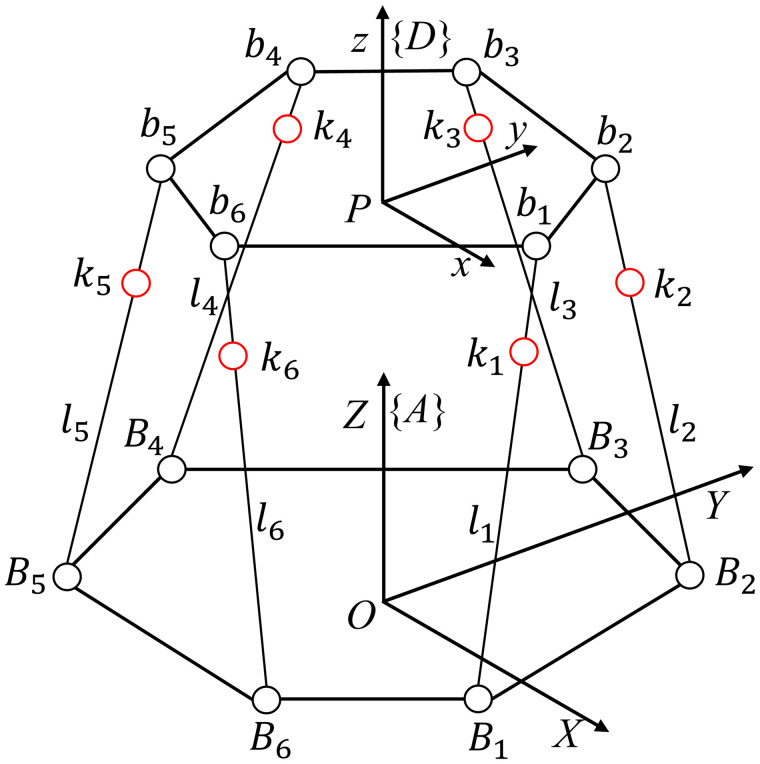
Schematic of the FSP.

In terms of structural configuration compared to the traditional rigid Stewart platform, the FSP innovatively incorporates piezoelectric ceramic stacks at the connection interface between the telescopic legs and the moving platform. In Fig 1, $b_{i}$ denotes the spherical joint on the moving platform, $B_{i}$ represents the spherical joint on the fixed platform, $l_{i}$ indicates the telescopic leg, and$k_{i}$refers to the piezoelectric ceramic stack.

**Fig 2:**
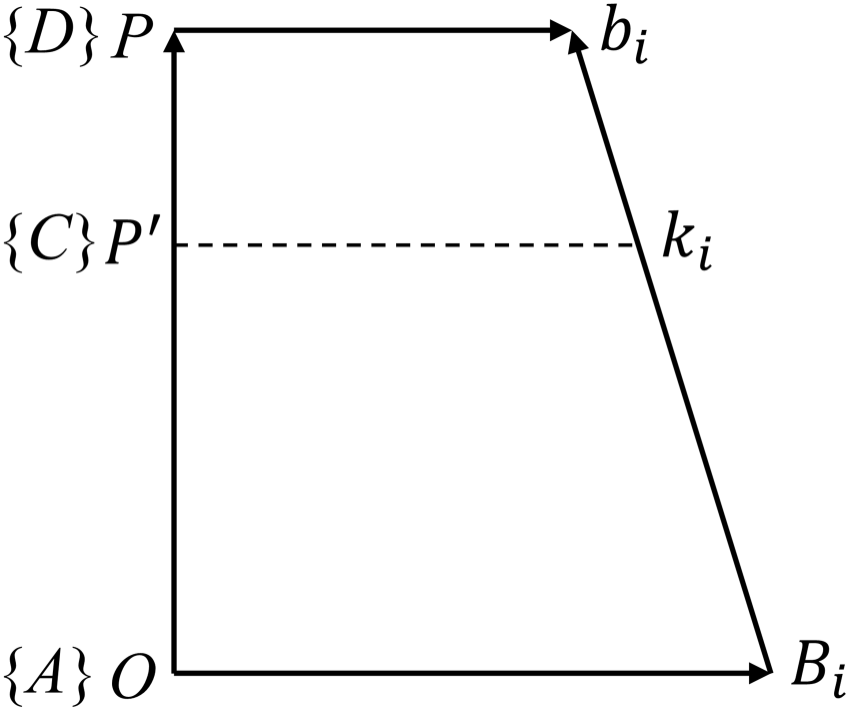
2D schematic of the FSP.

For the FSP, establish a coordinate system as shown in Fig 1. A two-dimensional representation is provided in Fig 2. For the i-th telescopic rod, a coordinate system $P^{\prime }uvw$ is set at the horizontal position corresponding to the piezoelectric ceramic $k_{i}$, denoted as {C}.

The column matrix POA=[@ccc@XPYPZP]T of the position vector $P_{O}$ of the origin P of the moving coordinate system Pxyz in the fixed coordinate system OXYZ; the column matrix POA′=[@ccc@XP′YP′ZP′]T of the position vector ${P_{O}}^{\prime }$ of the origin $P^{\prime }$ of the coordinate system $P^{\prime }uvw$ in the fixed coordinate system OXYZ; and the rotation transformation matrix of the moving coordinate system Pxyz relative to the fixed coordinate system OXYZ is:


RAD=(@ccc@CψCϕ−SψCθSϕ−CψSϕ−SψCθCϕSψSθSψCϕ+CψCθSϕ−SψSϕ+CψCθCϕ−CψSθSθSϕSθCϕCθ) (1)

In the formula, $C_{\psi }=cos\psi$,$C_{\phi }=cos\phi$,$C_{\theta }=cos\theta$,$S_{\psi }=sin\psi$, $S_{\phi }=sin\phi$, $S_{\theta }=sin\theta$, ψ is the precession angle, θ is the nutation angle, and ϕ is the spin angle.

The rotation transformation matrix of coordinate system $P^{\prime }uvw$ relative to the fixed coordinate system OXYZ is RAD. Since coordinate system {C} remains parallel to coordinate system {D}, the rotation matrix is RAC=RAD. The column matrix representing the position of the length vector $k_{i}$ of the i-th telescopic rod piezoelectric ceramic actuator $k_{i}$ in coordinate system $P^{\prime }uvw$ is kiC=[@ccc@ukivkiwki]T. The column matrix representing the position of the length vector $b_{i}$ of the i-th spherical hinge $b_{i}$ in the moving coordinate system Pxyz is biD=[@ccc@xbiybizbi]T. The column matrix representing the position of the length vector $B_{i}$ of the i-th spherical hinge $B_{i}$ in the fixed coordinate system OXYZ is POA=[@ccc@XBiYBiZBi]T. Then, the length vector of the i-th telescopic rod $B_{i}k_{i}$ is:


Biki=POA′+RACkiC−BiA (2)

The length vector of the i-th telescopic rod part $k_{i}b_{i}$ is:


kibi=(POA−POA′)+RADbiD−RACkiC (3)

The length vector of the i-th telescopic rod $B_{i}b_{i}$ is:


$$\begin{array}{c} L_{i}=B_{i}k_{i}+k_{i}b_{i} \end{array}$$ (4)

Assuming that $B_{i}k_{i}={\left[\begin{array}{ccc} @ccc@X_{1i} & Y_{1i} & Z_{1i} \end{array}\right]}^{T}$,$k_{i}b_{i}={\left[\begin{array}{ccc} @ccc@X_{2i} & Y_{2i} & Z_{2i} \end{array}\right]}^{T}$, Then the length of the i-th telescopic rod $l_{1i}$ is:


$$\begin{array}{c} l_{1i}=\sqrt{{X_{1i}}^{\text{2}}+{Y_{1i}}^{\text{2}}+{Z_{1i}}^{\text{2}}} \end{array}$$ (5)

The PEA compensation displacement of the i-th telescopic rod $l_{2i}$ is:


$$\begin{array}{c} l_{2i}=\sqrt{{X_{2i}}^{\text{2}}+{Y_{2i}}^{\text{2}}+{Z_{2i}}^{\text{2}}} \end{array}$$ (6)

The total length of the i-th telescopic rod $B_{i}b_{i}$ is:


$$\begin{array}{c} l_{i}=l_{1i}+l_{2i}=\sqrt{{X_{1i}}^{\text{2}}+{Y_{1i}}^{\text{2}}+{Z_{1i}}^{\text{2}}}+\sqrt{{X_{2i}}^{\text{2}}+{Y_{2i}}^{\text{2}}+{Z_{2i}}^{\text{2}}} \end{array}$$ (7)

To calculate the piezoelectric ceramic compensation displacement $l_{2i}$, it is necessary to first construct the Jacobian matrix [16–18] to establish the mapping relationship between the rod forces and the external forces acting on the moving platform:


$$\begin{array}{c} J=(\begin{array}{cc} \overset{T}{\underset{1}{s}} & {\left(r_{\text{1}}\times s_{\text{1}}\right)}^{T}\\ \overset{T}{\underset{2}{s}} & {\left(r_{\text{2}}\times s_{\text{2}}\right)}^{T}\\ \vdots & \vdots \\ \overset{T}{\underset{6}{s}} & {\left(r_{\text{6}}\times s_{\text{6}}\right)}^{T} \end{array}) \end{array}$$ (8)

where $s_{i}$ is the unit direction vector of the telescopic rod si=LiA/li, and $r_{i}$ is the vector from the hinge point $b_{i}$ of the motion platform to the center point P of the motion platform ri=RADbiD.

Let m be the mass of the moving platform and the load, and let g be the gravitational acceleration, taken as $9.8m/s^{\text{2}}$. Then, the gravity of the moving platform is given by:


$$\begin{array}{c} F_{m}=m\cdot g \end{array}$$ (9)

For the applied external force $F_{ext}={\left(\begin{array}{cccc} @cccc@\begin{array}{cc} \begin{array}{cc} F_{x} & F_{y} \end{array} & F_{z} \end{array} & M_{x} & M_{y} & M_{z} \end{array}\right)}^{T}$, $F_{x}$, $F_{y}$,and $F_{z}$ represent the projections of all telescopic leg axial forces onto the fixed coordinate system OXYZ, while $M_{x}$, $M_{y}$, and $M_{z}$ denote the projections of the moments exerted by the external force on the centerPof the moving platform onto the fixed coordinate system OXYZ. Since the moving platform is subjected to a normal force, we can obtain:


$$\begin{array}{c} F_{ext}={\left(\;\;\;\;\;\;\begin{array}{cccc} @cccc@\begin{array}{cc} \begin{array}{cc} 0 & 0 \end{array} & F_{m} \end{array} & 0 & 0 & 0 \end{array}\right)}^{T} \end{array}$$ (10)

Subsequently, it is necessary to transform the external forces acting on the moving platform into the axial forces of each telescopic leg. Through static analysis [19,20], the following can be obtained:


$$\begin{array}{c} F_{ext}=J^{T}\cdot f \end{array}$$ (11)

According to inverse statics, we have:


$$\begin{array}{c} f=J^{-T}\cdot F_{ext} \end{array}$$ (12)

where $f={\left(\begin{array}{cccc} @cccc@\begin{array}{cc} \begin{array}{cc} f_{\text{1}} & f_{\text{2}} \end{array} & f_{\text{3}} \end{array} & f_{\text{4}} & f_{\text{5}} & f_{\text{6}} \end{array}\right)}^{T}$, and $f_{i}$ denote the axial forces of the six telescopic legs, respectively.

The axial load borne by the piezoelectric ceramic on a single leg is exactly equal to the axial force $f_{i}$ of that telescopic leg. This axial load causes axial elastic compression deformation of the piezoelectric ceramic, thereby resulting in the displacement loss of the telescopic leg. The displacement loss of the Stewart platform during motion refers to the axial displacement loss generated to overcome the gravity of the moving platform and the load during the movement of the telescopic legs. This is equivalent to the pure elastic deformation $l_{2i}$ of the piezoelectric ceramic stack under axial external force, which can be calculated using Hooke’s Law for axial tension and compression:


$$\begin{array}{c} l_{2i}=(f_{i}\cdot h)/(E\cdot A) \end{array}$$ (13)

where h is the thickness of the piezoelectric ceramic stack, Eis the elastic modulus of the piezoelectric ceramic stack, and A is the cross-sectional area of the piezoelectric ceramic stack.

### Disturbance modeling and actuator characteristic analysis

To verify the engineering practicality of the piezoelectric ceramic displacement compensation method proposed in this paper, it is necessary to analyze the influence of external disturbances on the actuator control input and saturation characteristics. During the actual operation of the Stewart platform, it is mainly subjected to three types of disturbances: load mass fluctuation disturbance, inertial force disturbance generated by platform motion, and external unknown shock disturbance. By introducing the disturbance term into the system statics model, the total external force can be obtained as:


$$\begin{array}{c} F_{total}=F_{ext}+F_{d} \end{array}$$ (14)

where $F_{d}$ denotes the external disturbance load.

According to the principle of inverse statics, the disturbance load is mapped into the axial force variations of each telescopic leg via the Jacobian matrix:


$$\begin{array}{c} f=J^{-T}\cdot F_{total} \end{array}$$ (15)

According to Hooke’s law for axial tension and compression of piezoelectric ceramics and the inverse piezoelectric effect, the compensation displacement and the required driving voltage are respectively:


$$\begin{array}{c} \Delta l=(f_{i}\cdot h)/(E\cdot A) \end{array}$$ (16)

$$\begin{array}{c} U=\Delta l/d_{33} \end{array}$$ (17)

where $d_{33}$ is the piezoelectric coefficient.

## Analysis of forward kinematics

### Analysis of the non-singular matrix trap in Newton’s iteration method

In the forward kinematics of the Stewart platform, the core formula of the traditional Newton’s iteration method is:


$$\begin{array}{c} X_{k+1}=X_{k}+\delta X_{k} \end{array}$$ (18)

where the iterative correction term is:


$$\begin{array}{c} \delta X_{k}=-J^{+}\left(X_{k}\right)\cdot F\left(X_{k}\right) \end{array}$$ (19)

where $X_{k}$ is the pose estimate of the moving platform at the k-th iteration, $F\left(X_{k}\right)$ is the leg length residual vector at the k-th iteration, $J\left(X_{k}\right)$ is the Jacobian matrix at the k-th iteration, and $J^{+}$ is the pseudo-inverse of the Jacobian matrix.

When solving the forward kinematics of the Stewart platform using the traditional Newton’s iteration method, a phenomenon may occur in certain regions of the workspace where the Jacobian matrix is full-rank, yet the iteration fails to converge. This paper terms this phenomenon the “non-singular matrix trap.” The fundamental cause is that the Jacobian matrix exhibits severe ill-conditioning at specific poses, leading to numerical instability. The numerical stability of the Stewart platform’s Jacobian matrix is quantified by the condition number as follows:


$$\begin{array}{c} cond\left(J\right)=\sigma_{max}\left(J\right)/\sigma_{min}\left(J\right) \end{array}$$ (20)

where $\sigma_{max}$ and $\sigma_{min}$ are the maximum and minimum singular values of the Jacobian matrix, respectively.

The larger the condition number, the stronger the amplification effect on input errors, and the worse the numerical stability. When $cond\left(J\right)>10^{\text{3}}$, the matrix enters the ill-conditioned range; when $cond\left(J\right)>10^{\text{5}}$, it exhibits severe ill-conditioning. At this point, although the Jacobian matrix remains mathematically full-rank (non-singular) and does not trigger a rank-deficiency error, the pseudo-inverse $J^{+}$ will still drastically amplify errors. This leads to numerical instability in the iterative correction term $\delta X_{k}$, ultimately causing convergence failure.

### BP neural network architecture

**Fig 3:**
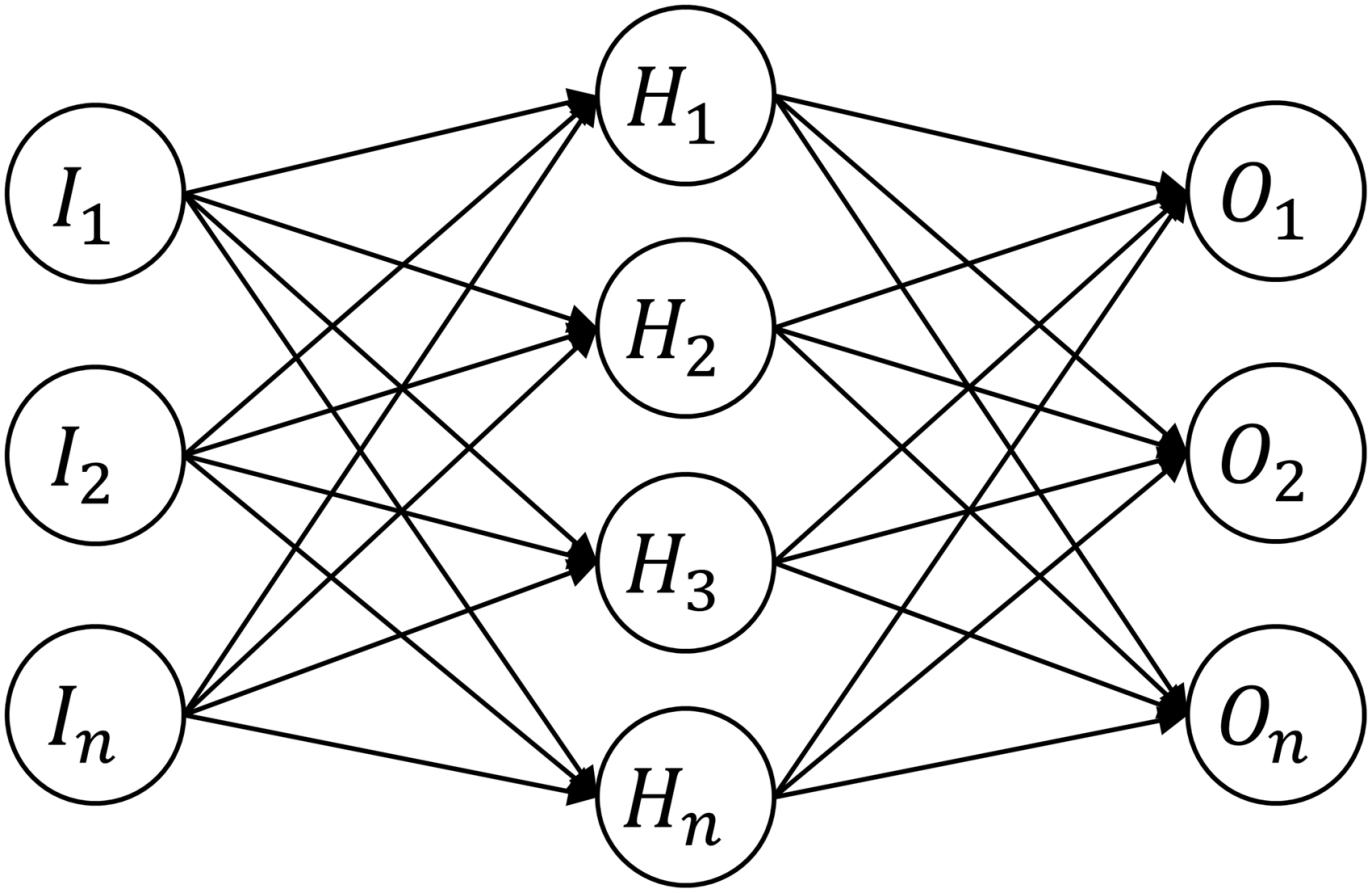
Structure of the BP neural network.

The BP neural network [21] is a multi-layer feedforward neural network trained via the error backpropagation algorithm. As illustrated in Fig 3, it consists of an input layer *I*, a hidden layer *H*, and an output layer *O*. Each layer contains several neurons connected by weights. Its working process is divided into two phases: signal forward propagation and error backpropagation. During forward propagation, input signals travel from the input layer to the output layer via the hidden layer. Conversely, during backpropagation, output errors are transmitted back along the original path, and network parameters are adjusted using the gradient descent method. This bidirectional mechanism endows the network with strong nonlinear fitting capabilities, allowing it to directly establish the mapping relationship between inputs and outputs. Consequently, it can efficiently handle complex nonlinear problems, providing a more reliable solution for the forward kinematics calculation of the FSP.

### Improvement of BP neural network based on L-M algorithm

As a widely used nonlinear least-squares optimization method, the L-M algorithm combines the fast convergence of Newton’s method with the stability of traditional BP neural networks, demonstrating superior convergence speed and lower mean square error. The L-M algorithm achieves a low-cost approximation of the Hessian matrix through the Jacobian matrix J, expressed as $H\approx J^{-T}J$. Meanwhile, it introduces an adaptive damping factor λ to modify the iteration formula. The final iteration formula is given by:


$$\begin{array}{c} \Delta \omega =-{\left(J^{T}J+\lambda I\right)}^{-1}\cdot J^{-T}e \end{array}$$ (21)

When λ is large, the λI term dominates the matrix inversion, causing the iteration formula to degenerate into the first-order gradient descent method. In this case, the algorithm step size is small, which ensures global convergence and avoids iteration oscillation and divergence.

When λ is small, the influence of the λI term becomes negligible, and the iteration formula degenerates into the Gauss-Newton method. In this case, the algorithm fully utilizes second-order curvature information to adjust the iteration direction and step size, thereby retaining the rapid quadratic convergence.

In the actual training process, the algorithm automatically updates the value of λ based on the error variation in each epoch: if the loss function value decreases in the current iteration, λ is reduced to accelerate the convergence speed; if the loss function value increases, λ is increased to ensure iteration stability.

**Fig 4:**
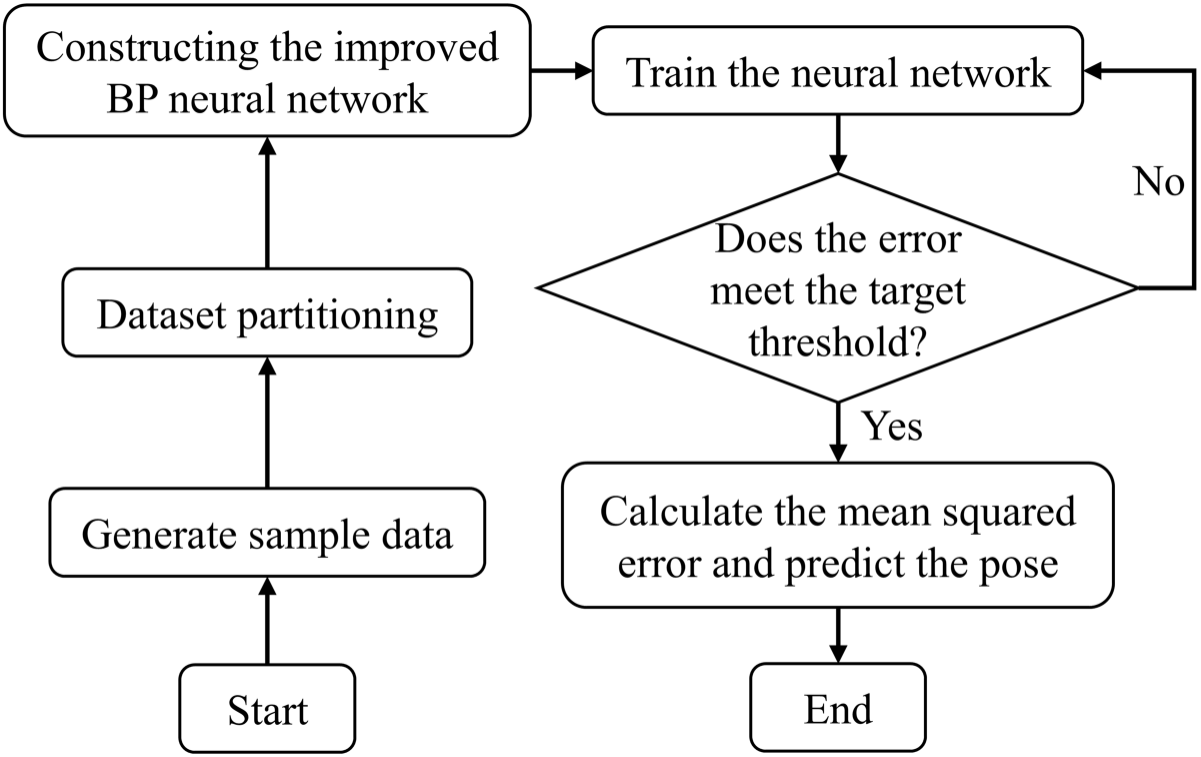
Flowchart of the improved BP neural network method based on the L-M algorithm.

Based on this, this study innovatively proposes a kinematic forward solution method that combines the L-M algorithm with a BP neural network. By embedding the L-M algorithm into the training process of the BP neural network, this method effectively overcomes the problems of slow convergence speed and low solving efficiency encountered by traditional BP networks in solving the forward kinematics of flexible Stewart platforms. It provides a new approach that combines theoretical innovation with engineering practicality for the efficient solution of flexible Stewart platform kinematics. The flowchart of the improved BP neural network method based on the L-M algorithm is shown in Fig 4.

### Network parameter settings and procedure

The hidden layer structure of a BP neural network directly determines its nonlinear fitting capability and generalization performance. An insufficient number of neurons leads to underfitting, failing to capture the complex mapping relationship between input rod lengths and output poses; conversely, an excessive number increases model complexity, thereby causing overfitting and reducing training efficiency. Regarding single-hidden-layer BP neural networks, there is currently no strict theoretical formula to directly determine the optimal number of neurons. Therefore, this paper adopts a two-stage optimization strategy combining heuristic preliminary screening and grid search verification to determine the network structure. The heuristic preliminary screening process is as follows:

The reasonable range for the number of hidden layer neurons can be estimated by the following equation:


$$\begin{array}{c} H_{n}\approx \sqrt{I_{n}+O_{n}}+\alpha \end{array}$$ (22)

where α is an adjustment constant, typically taking an integer value between 0 and 10.

In the forward kinematics problem of the Stewart platform discussed in this paper, the inputs are the lengths of the six driving rods ($I_{n}=6$), and the outputs are the 6-DOF pose parameters of the moving platform ($O_{n}=6$). The search range is expanded to [10, 50] to cover all possible optimal configurations. On this basis, the grid search method is employed to quantitatively evaluate all candidate configurations within this range. A step size of 10 is used to traverse five neuron configurations [10, 20, 30, 40, 50]. Under each configuration, identical training parameters are utilized, and the Mean Square Error (MSE) of the validation set serves as the performance metric. The formula for calculating the validation set MSE is given by:


$$\begin{array}{c} MSE=\frac{\text{1}}{N}\sum_{i=1}^{N}\frac{\text{1}}{\text{6}}{\sum_{j}^{\text{6}}\left(y_{ij}-\tilde{y_{ij}}\right)}^{\text{2}} \end{array}$$ (23)

where N denotes the number of validation samples, $y_{ij}$ represents the true pose parameter of the j-th dimension for the i-th sample, and $\tilde{y_{ij}}$ is the corresponding predicted pose parameter.

**Fig 5:**
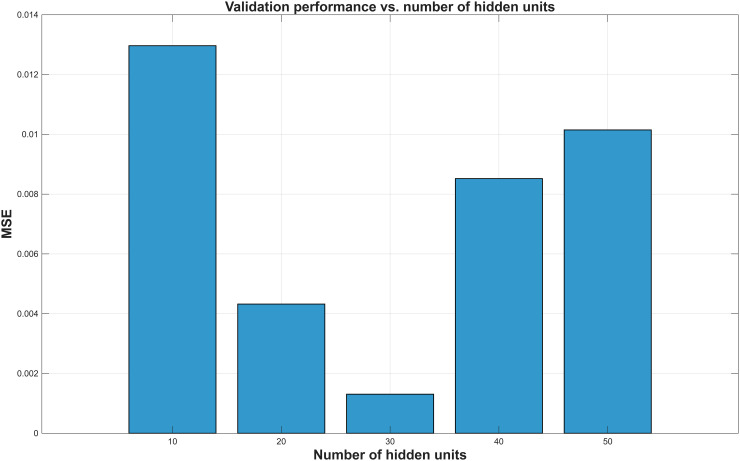
Validation performance vs. number of hidden units.

As shown in Fig 5, the validation set MSE reaches a minimum value of 0.001307 when the number of hidden layer neurons is 30. When the number of neurons is further increased to 40 and beyond, the validation MSE no longer decreases significantly; instead, it exhibits a slight upward trend, indicating that the model begins to suffer from overfitting. Therefore, this paper ultimately determines to adopt a BP neural network structure with a single hidden layer and 30 neurons for the forward kinematics solution of the Stewart platform.

**Fig 6:**
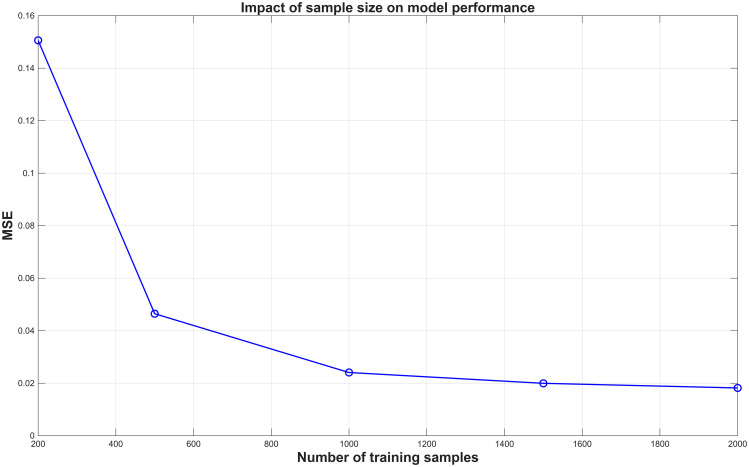
Ablation study.

To determine the optimal sample size for training the neural network, an ablation study on sample size was conducted. Five different training sample sizes were set: 200, 500, 1000, 1500, and 2000. For each group, samples were uniformly and randomly generated within the nominal workspace of the Stewart platform. All models utilized the same network architecture and training parameters. The data was split into training, validation, and testing sets in a 7:1:2 ratio. Using the Mean Squared Error (MSE) on the test set as the performance metric, the sample size of 2000 was ultimately selected as it yielded the minimum MSE. The ablation study is illustrated in Fig 6.

**Fig 7:**
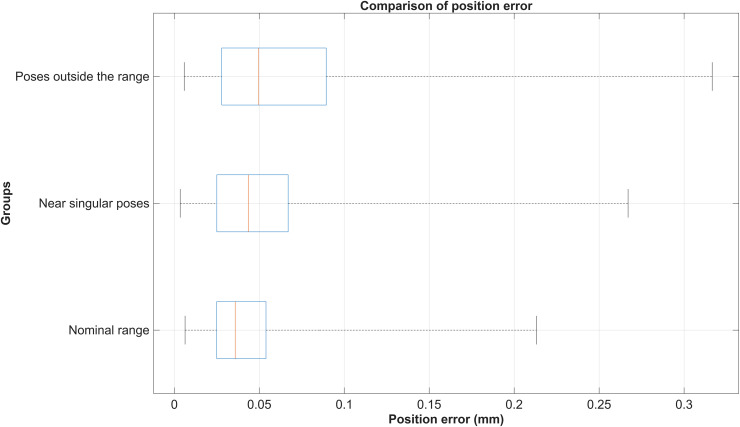
Comparison of position errors.

**Fig 8:**
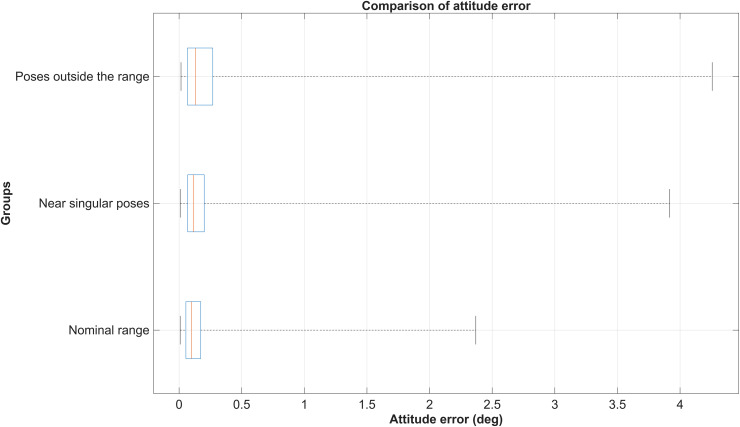
Comparison of attitude errors.

To verify the generalization ability of the model outside the nominal workspace, especially its performance near singular poses and beyond the nominal sampling range, three independent test sets are constructed in this paper, each containing 200 new samples. The first is the nominal range test set, whose sampling range is completely consistent with that of the training set, to evaluate the basic accuracy of the model within the training range. The second is the test set near singular poses, where the sampling range is extended to the boundary of the workspace to cover singular regions with a sharp variation rate of rod length: x∈[−11,0]mm, y∈[−11,0]mm, z∈[295,306]mm, $\psi ,\theta ,\phi \in \left[0,5{\mathrm{.1}}^{\circ }\right]$. The third is the out-of-range pose test set, with the sampling range further exceeding the nominal range, for evaluating the generalization ability of the model in completely unfamiliar regions: x∈[−12,0]mm, y∈[−12,0]mm, z∈[295,307]mm, $\psi ,\theta ,\phi \in \left[0,5{\mathrm{.2}}^{\circ }\right]$. The comparisons of positional error and attitude error are presented in Fig 7 and Fig 8, respectively.

Simulation results show that the position and attitude errors of the model near singular poses only increase slightly compared with those in the nominal range, and the overall accuracy remains at a high level. In regions beyond the nominal range, although the model errors rise to a certain extent, they are still within the acceptable range for engineering applications. The error distribution is relatively concentrated without severe accuracy degradation. It is fully demonstrated that the trained neural network not only maintains stable prediction accuracy within the nominal workspace, but also possesses excellent generalization ability, which can be applied to pose prediction tasks near singular poses and outside the nominal range.

This paper employs a BP neural network improved by the Levenberg-Marquardt (L-M) algorithm to solve the forward position and orientation of the Stewart platform. The experimental procedure is as follows:

(1) Based on the inverse kinematics model of the Stewart platform, 2,000 non-repetitive samples are generated within its effective workspace using uniform random sampling. Each sample consists of the lengths of the six legs as inputs and the corresponding 6-DOF pose as outputs.
(2) After shuffling all samples, they are divided into training, validation, and testing sets in a ratio of 7:1:2. Unlike the commonly used 8:1:1 split in academia, this study increases the proportion of the testing set. The core rationale is that our primary goal is to verify the model’s prediction accuracy and robustness in real-world scenarios with unknown poses. A more substantial testing set allows for a more comprehensive and rigorous examination of model performance, ensuring that the reported metrics truly reflect actual working effectiveness. Meanwhile, the 70% allocation for training remains sufficient to meet the fitting needs of the neural network without causing underfitting.
(3) A single-hidden-layer BP neural network is constructed with an input layer dimension of 6 (corresponding to the six leg lengths), 30 neurons in the hidden layer, and an output layer dimension of 6 (corresponding to the 6-DOF pose). The training function employs the L-M algorithm, chosen for its faster convergence and higher fitting accuracy. The maximum number of training iterations is set to 1,000, the initial learning rate is 0.01, and the training target Mean Square Error (MSE) is 1 × 10 ⁻ ⁴.
(4) All input and output data are normalized. The network is trained using the training set, while the validation set is used to monitor the process in real-time via the toolbox’s built-in early stopping mechanism. Training is automatically terminated, and optimal model parameters are saved when the validation error fails to decrease consecutively, effectively preventing overfitting.
(5) The Mean Square Error (MSE) of the model is calculated based on the testing set to evaluate its generalization performance. The trained model can directly input leg length data and rapidly output the corresponding predicted platform pose.

This study is conducted within the MATLAB environment, utilizing the feedforwardnet function from the Deep Learning Toolbox to construct a single-hidden-layer BP neural network for solving the inverse kinematics of the Stewart platform. The model features a single hidden layer with 10 neurons, employing a hyperbolic tangent sigmoid function as the activation function, while the output layer utilizes a linear activation function (purelin) to ensure continuous output of pose parameters. Network weights and biases are initialized via the Nguyen-Widrow algorithm, which distributes the active regions of hidden layer neurons evenly across the input sample range to accelerate convergence. The training process employs the Levenberg-Marquardt algorithm with a learning rate of 0.01, a maximum of 1,000 iterations, and a target training error of 1 × 10 ⁻ ⁶. The Mean Square Error (MSE) serves as the loss function to minimize the deviation between predicted and actual poses. Overfitting is primarily controlled through a streamlined network architecture and an early stopping mechanism; specifically, the validation set error is monitored in real-time during training, and the process is terminated early to preserve optimal parameters when the validation error fails to decrease consecutively. A total of 2,000 rod length-pose sample pairs were randomly generated and split into training, validation, and testing sets in a 7:1:2 ratio for model training and performance evaluation.

## Simulation verification

**Fig 9:**
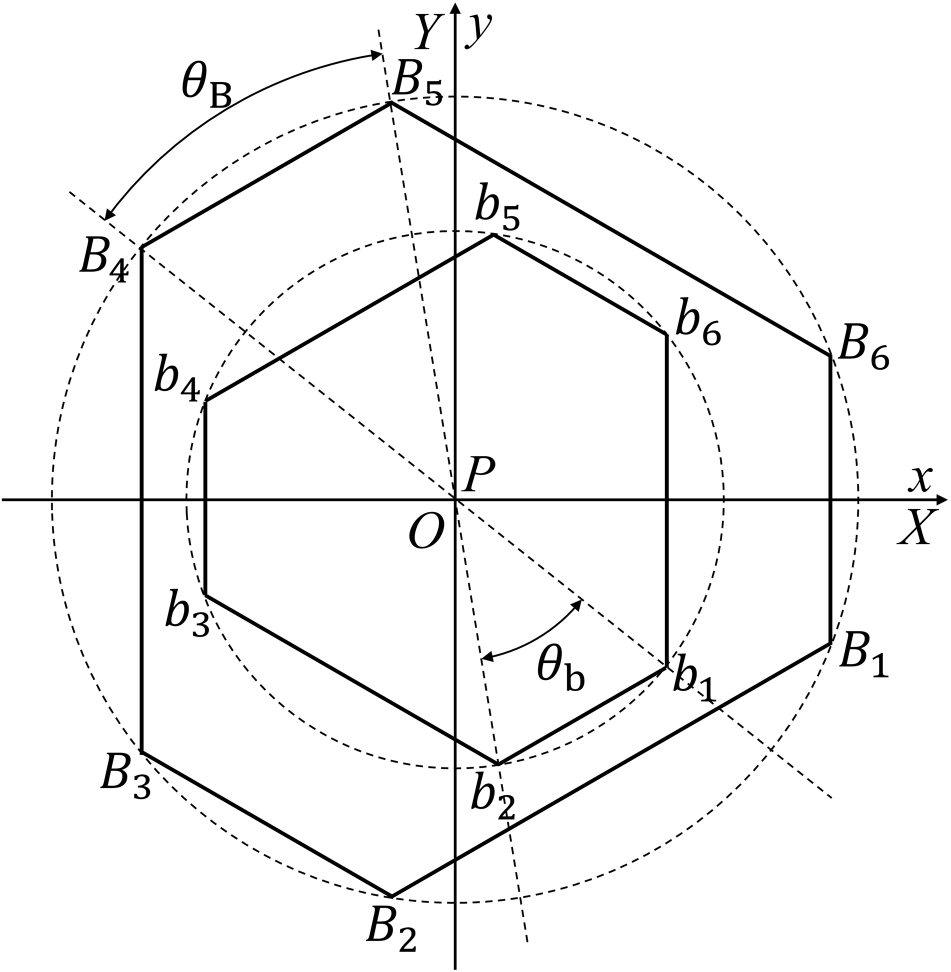
Distribution of FSP Hinge Points.

The platform parameters are listed in Table 1. By rationally determining the spatial coordinates of the hinge points on both the moving and fixed platforms, an accurate initial pose model of the FSP can be established, providing a reliable benchmark for subsequent simulation verification. The distribution of the platform hinge points is illustrated in Fig 9, and their specific coordinates are detailed in Table 2.

**Table 1:** Platform Parameters.

Parameter	Dimension	Parameter	Dimension
r **(mm)**	120	ψ **(°)**	2
R **(mm)**	200	θ **(°)**	3
$\theta_{b}$**/**$\theta_{B}$ **(°)**	30	ϕ **(°)**	4
$X_{P}$ **(mm)**	−5	h **(mm)**	10
$Y_{P}$ **(mm)**	−5	E **(Pa)**	5 × 10^10^
$Z_{P}$ **(mm)**	300	A **(mm²)**	452.39

**Table 2:** Coordinates of FSP Hinge Points.

Moving Platform	$x_{bi}$(mm)	$y_{bi}$(mm)	$z_{bi}$(mm)	Fixed Platform	$X_{Bi}$(mm)	$Y_{Bi}$(mm)	$Z_{Bi}$(mm)
$b_{\text{1}}$	84.8528	−84.8528	0.0000	$B_{\text{1}}$	193.1852	−51.7638	0.0000
$b_{\text{2}}$	31.0583	−115.9111	0.0000	$B_{\text{2}}$	−51.7638	−193.1852	0.0000
$b_{\text{3}}$	−115.9111	−31.0583	0.0000	$B_{\text{3}}$	−141.4214	−141.4214	0.0000
$b_{\text{4}}$	−115.9111	31.0583	0.0000	$B_{\text{4}}$	−141.4214	141.4214	0.0000
$b_{\text{5}}$	31.0583	115.9111	0.0000	$B_{\text{5}}$	−51.7638	193.1852	0.0000
$b_{\text{6}}$	84.8528	84.8528	0.0000	$B_{\text{6}}$	193.1852	51.7638	0.0000

### Inverse kinematics simulation

**Fig 10:**
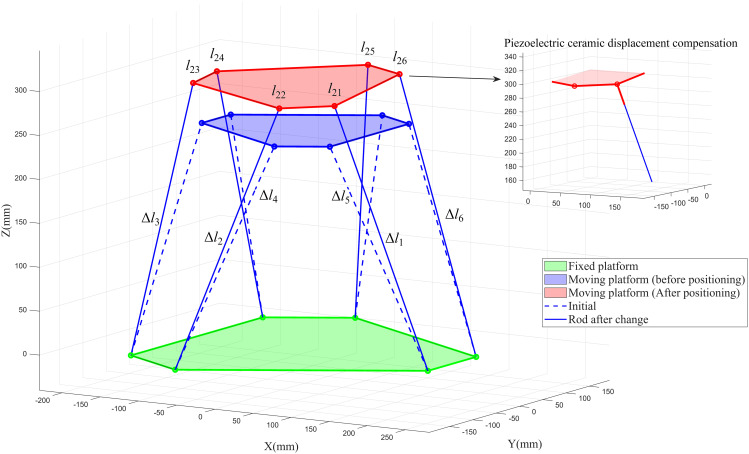
Overall View of the FSP.

By inputting the initial pose parameters of the FSP listed in Table 1, and utilizing the FSP inverse pose solution model alongside the MATLAB simulation platform, the simulation results for each component of the FSP were obtained. The overall view of the FSP is shown in Fig 10, and the specific data are detailed in Table 3.

**Table 3:** Simulation Results of FSP Components.

Parameter	$l_{1}$(mm)	$l_{2}$(mm)	$l_{3}$(mm)	$l_{4}$(mm)	$l_{5}$(mm)	$l_{6}$(mm)
$l_{\text{0}}$	274.464534	274.464534	274.464534	274.464534	274.464534	274.464534
Δl	40.775158	42.320942	38.759054	53.168150	48.652343	56.042905
$l_{i}$	315.239693	316.785476	313.223588	327.632684	323.116878	330.507440
$l_{2i}$	40.831 × 10 ⁻ ^6^	36.657 × 10 ⁻ ^6^	47.089 × 10 ⁻ ^6^	27.970 × 10 ⁻ ^6^	47.417 × 10 ⁻ ^6^	31.280 × 10 ⁻ ^6^

The simulation results indicate that the loss displacement range of the proposed method covers 27.9 nm to 47.4 nm. The results clearly present the extension variations of each telescopic leg during the high-precision positioning process of the moving platform, and successfully solve for the $l_{2i}$ under given load conditions, thereby achieving high-precision positioning.

### Finite element simulation of piezoelectric ceramics

**Fig 11:**
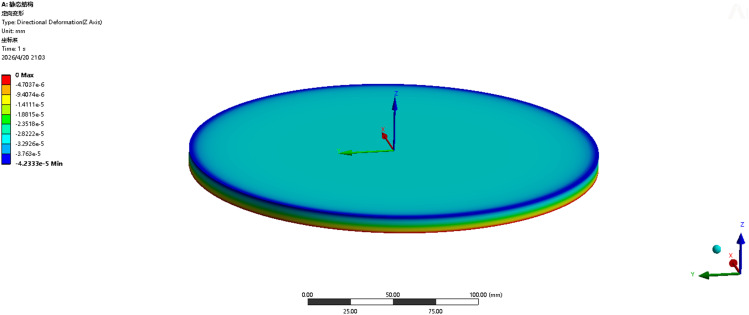
Finite Element Simulation of Piezoelectric Ceramic Stack.

Finite element simulation of the piezoelectric ceramic stack was conducted using Ansys software. For telescopic leg 1, the deformation of the mounted piezoelectric ceramic stack under axial force is shown in Fig 11.

As shown in Fig 8, the Ansys simulation result is 42.333 × 10 ⁻ ⁶ mm, and the relative error between this value and the theoretical calculation of 40.831 × 10 ⁻ ⁶ mm is 3.68%, which verifies the rationality of the proposed scheme.

### Comparative analysis of platform manipulability distribution and manipulability ellipsoids before and after compensation

Manipulability is a core metric for evaluating the motion and force transmission performance of parallel mechanisms. It is defined as the square root of the determinant of the product of the Jacobian matrix and its transpose:


$$\begin{array}{c} \omega =\sqrt{det\left(JJ^{T}\right)} \end{array}$$ (24)

**Fig 12:**
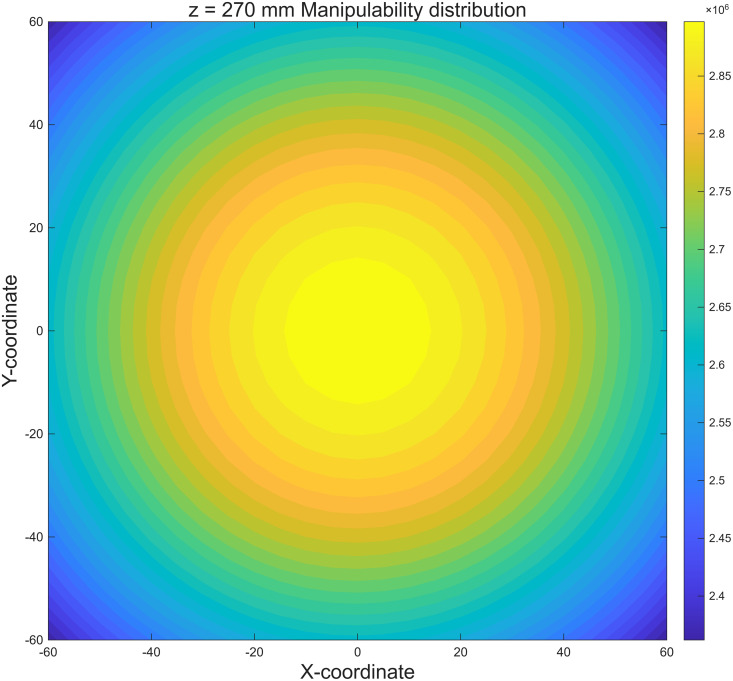
Manipulability Distribution Contour Map at a Moving Platform Height of 270 mm.

A larger manipulability value indicates better motion flexibility and higher positioning accuracy of the platform. Conversely, when the manipulability approaches zero, the platform is in a singular configuration and cannot move normally. The manipulability distribution contour map at a moving platform height of 270 mm is shown in Fig 12.

As shown in Fig 12, the manipulability distribution exhibits distinct centro-symmetric characteristics: the manipulability is highest near the central pose, where the platform achieves optimal motion and force transmission performance. As the moving platform moves towards the workspace boundary, the manipulability gradually decreases, leading to continuous performance degradation. Notably, there are no regions where manipulability approaches zero within the entire workspace, indicating that the platform design is reasonable and free from singular configurations (lock-up) within the specified operating range.

**Fig 13:**
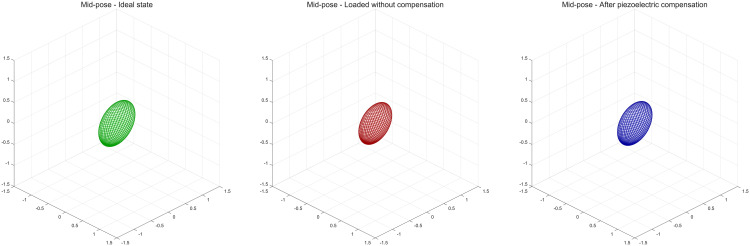
Comparison of Manipulability Ellipsoids at the Central Pose Before and After Compensation.

**Fig 14:**
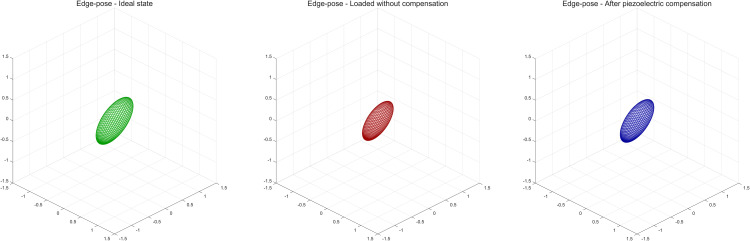
Comparison of Manipulability Ellipsoids at the Edge Pose Before and After Compensation.

Manipulability ellipsoids intuitively reflect the differences in the platform’s motion performance along different directions. The central pose and a typical edge pose were selected to comparatively analyze the manipulability ellipsoids under three conditions: the ideal state, the loaded state without compensation, and the state with piezoelectric compensation. The results are shown in Figs 13 and 14.

As shown in Fig 13, under the loaded state without compensation at the central pose, the volume of the manipulability ellipsoid decreases slightly and its shape undergoes minor distortion, indicating a certain degree of degradation in the platform’s overall performance. After piezoelectric ceramic displacement compensation, both the volume and shape of the ellipsoid recover to a state close to the ideal one. This demonstrates that the compensation scheme effectively eliminates load-induced displacement loss at the central pose and restores the platform’s ideal positioning accuracy.

As shown in Fig 14, under the loaded state without compensation at the edge pose, the manipulability ellipsoid volume decreases significantly and undergoes severe distortion, leading to a substantial degradation in platform performance. After piezoelectric ceramic displacement compensation, the volume increases markedly and the shape improves significantly, approaching the ideal state. A comparison between Fig 10 and Fig 11 reveals that the performance enhancement provided by the piezoelectric compensation scheme is more pronounced at the edge pose. This observation aligns with the displacement loss calculation results, fully verifying the effectiveness of the proposed compensation scheme in improving the accuracy of the platform’s weak links.

### Analysis of actuator characteristics and saturation boundaries under disturbance conditions

This paper discusses three disturbance conditions: The Nominal Condition without Disturbance, where only the rated load of 490 N is applied, serves as the baseline reference. The Step Load Disturbance Condition involves a sudden application of a 200 N axial load at t = 1 s to simulate a real-world scenario of sudden loading. The Sinusoidal Fluctuation Disturbance Condition applies a sinusoidal load with an amplitude of 150 N and a frequency of 1 Hz to simulate the inertial force fluctuations generated by the moving platform’s motion. The saturation boundary parameters of the piezoelectric stack are listed in Table 4.

**Table 4:** Saturation Boundary Parameters of the Piezoelectric Stack.

Parameter	Numerical values	Units
**Maximum Driving Voltage**	150	V
**Maximum Axial Force**	169.65	N
**Maximum Compensation Displacement**	75	nm

**Fig 15:**
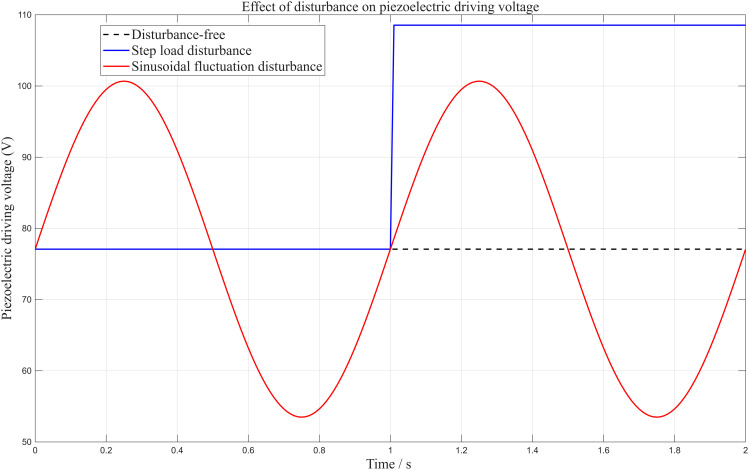
Effect of Disturbance on the Driving Voltage of Piezoelectric Ceramics.

As shown in Fig 15, under the nominal condition without disturbance, the average driving voltage of the piezoelectric ceramics is 77.1 V, which is only 51.4% of the maximum driving voltage, leaving a sufficient safety margin. When a 200 N step disturbance is applied, the driving voltage rises instantaneously to 108.5 V, which is still far below the saturation boundary of 150 V. Under sinusoidal fluctuation disturbance, the driving voltage fluctuates regularly around the rated value, with a peak of 100.7 V, also remaining within the saturation limit.

**Fig 16:**
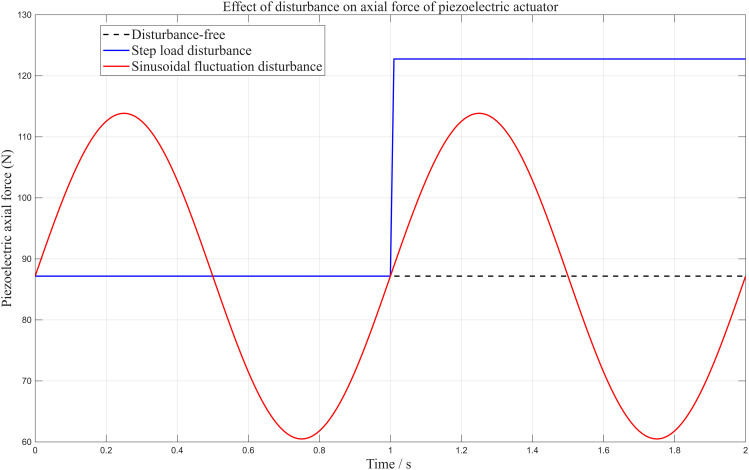
Effect of Disturbance on the Axial Force of Piezoelectric Ceramics.

Fig 16 presents the average axial force of the piezoelectric ceramics under different operating conditions. The average axial force is 81.2 N without disturbance, rises to 122.8 N after the step disturbance, and reaches a peak of 113.9 N under sinusoidal disturbance. All these values are lower than the maximum axial force limit of 169.65 N, indicating that the output capability of the piezoelectric ceramics fully meets the system requirements.

**Fig 17:**
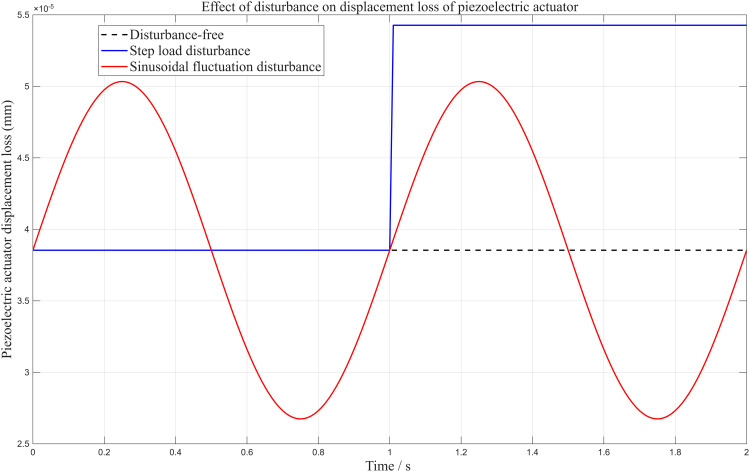
Effect of Disturbance on the Displacement Loss of Piezoelectric Ceramics.

Fig 17 illustrates the curves of compensation displacement under disturbances. The average compensation displacement is 38.5 nm without disturbance, increases to 54.3 nm after the step disturbance, and reaches a peak of 50.3 nm under sinusoidal disturbance. The compensation displacement varies linearly with the disturbance amplitude, which validates the correctness of the established static compensation model.

### Forward kinematics simulation

The training target error is assumed to be 1 × 10 ⁻ ⁶. The predicted initial pose parameters are shown in Table 5.

**Table 5:** Predicted Initial Pose.

Parameter	Actual Pose	Newton Iteration Input Predicted Pose	Traditional BP Input Predicted Pose	Improved BP Input Predicted Pose
$X_{P}$ **(mm)**	−5	−4.9	[−10, 0)	[−10, 0)
$Y_{P}$ **(mm)**	−5	−4.9	[−10, 0)	[−10, 0)
$Z_{P}$ **(mm)**	300	299.9	[295, 305)	[295, 305)
ψ **(°)**	2	1.9	[0, 5)	[0, 5)
θ **(°)**	3	2.9	[0, 5)	[0, 5)
ϕ **(°)**	4	3.9	[0, 5)	[0, 5)

**Fig 18:**
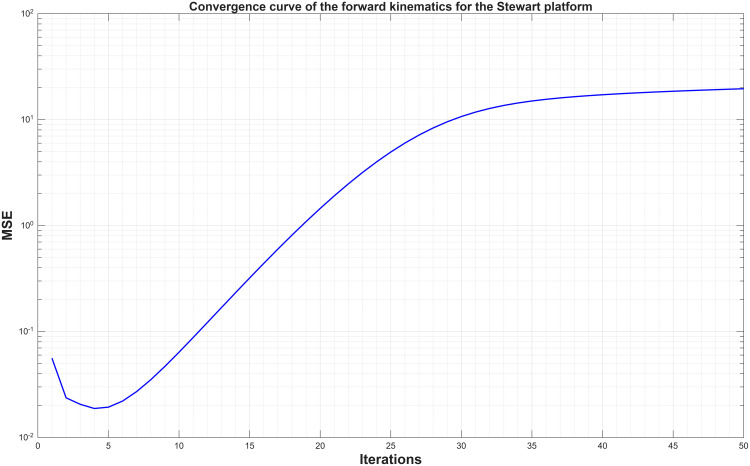
Convergence curve of the Newton-Raphson method.

First, the Newton-Raphson method exhibits significant limitations in solving the forward kinematics of 6-DOF parallel mechanisms: it is highly dependent on the initial value, and slight fluctuations in the initial value can easily cause the iteration process to fall into a non-singular matrix trap. Therefore, the predicted pose near the true value is used as the input, and its convergence curve is shown in Fig 18.

**Fig 19:**
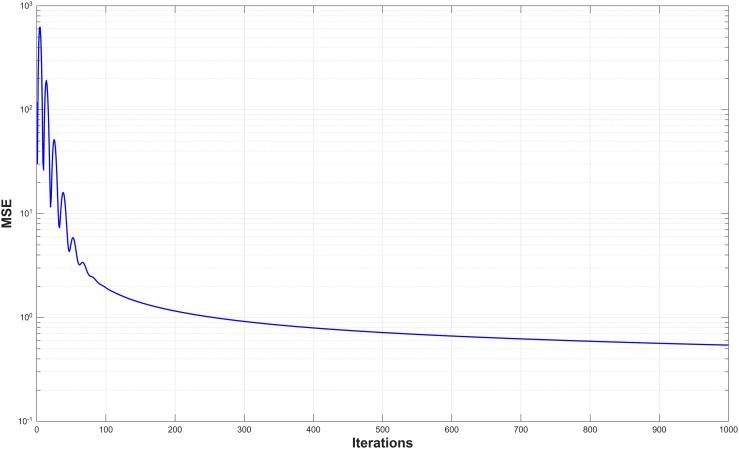
Convergence curve of the traditional BP neural network method.

**Fig 20:**
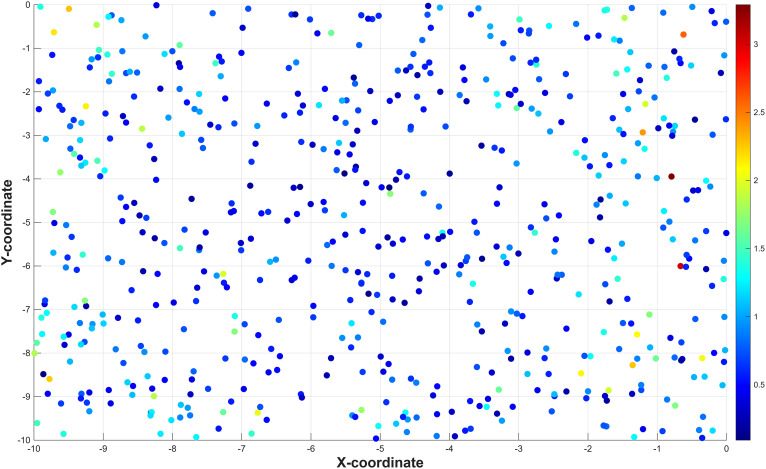
Heatmap of x-y plane position error for the traditional BP neural network.

Although the traditional BP neural network demonstrates significantly superior computational efficiency compared to the Newton-Raphson method and can accommodate data input ranges near the actual pose, it suffers from slow convergence speed, resulting in unsatisfactory overall solution efficiency. Furthermore, its mean squared error (MSE) performance on the test set is not ideal. Therefore, for the predicted pose with input near the actual pose range, the test set convergence curve is shown in Fig 19. The x-y plane position error heatmap is presented in Fig 20.

**Fig 21:**
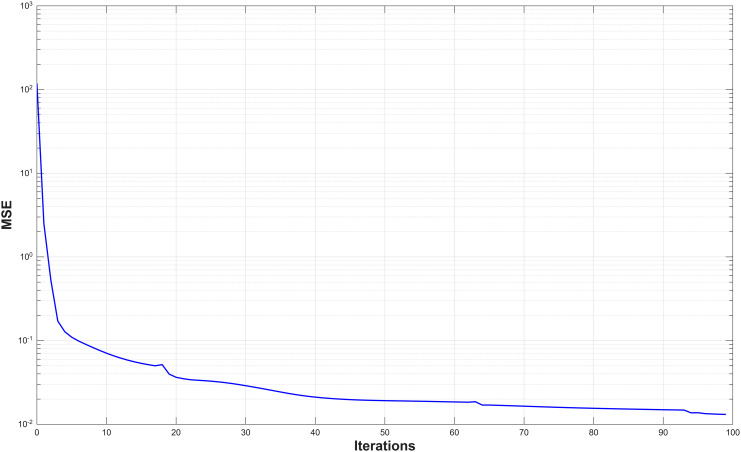
Convergence curve of the improved BP neural network method.

**Fig 22:**
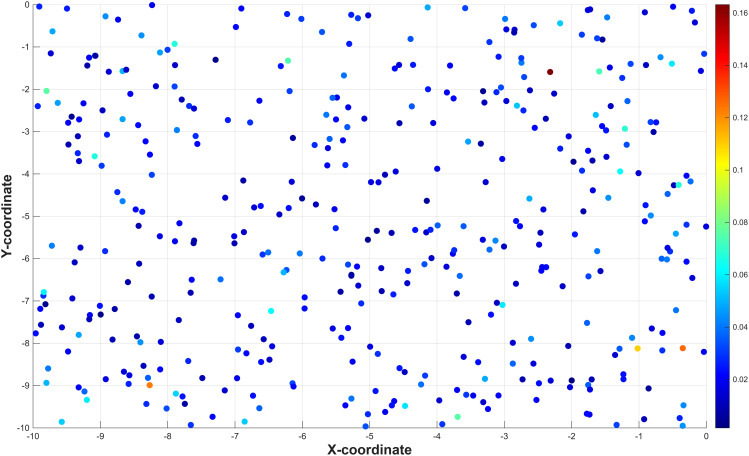
Heatmap of x-y plane position error for the improved BP neural network.

Embedding the L-M algorithm into the BP neural network to construct the nonlinear mapping relationship between rod length and pose parameters can effectively address the limitations of the Newton-Raphson method, as well as the problems of slow convergence speed and low solution efficiency in traditional BP neural networks. Furthermore, a smaller mean square error is achieved. The convergence curve of the test set is shown in Fig 21, and the x-y plane position error heatmap is presented in Fig 22.

**Fig 23:**
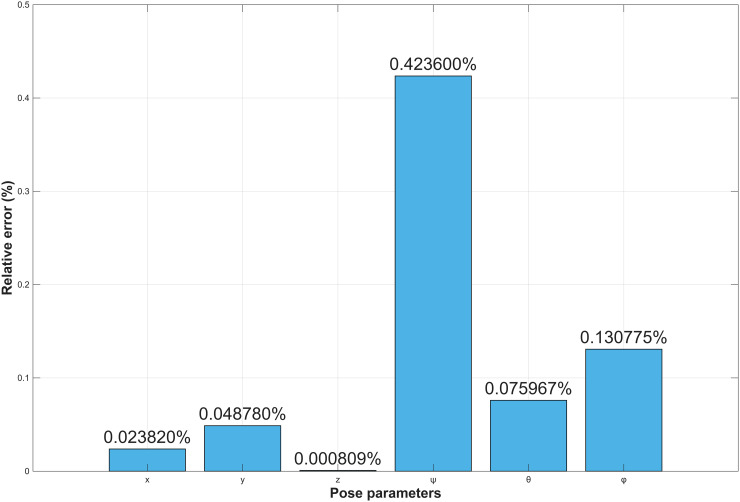
Relative error of each pose parameter.

Using the $l_{i}$ obtained from the inverse kinematics solution of the initial pose parameters as the input features of the BP neural network, the relative error between the predicted pose parameters and the initial pose parameters is shown in Fig 23.

The computational time of each forward kinematics solution method is shown in Table 6.

**Table 6:** Computational time.

Forward kinematics solution method	Computational time
**Newton-Raphson method**	1.0755 ms
**Traditional BP neural network method**	1.9387 ms
**Improved BP neural network method**	8.4152 ms

MATLAB simulation results demonstrate that when any set of inverse kinematics solutions within the FSP motion space is given as input features, the relative error of pose prediction by the proposed improved algorithm is globally controlled within 0.5%, with a minimum relative error as low as 8.09 × 10 ⁻ ⁴%. The absolute error in the heatmap is controlled around 0.02 mm, meeting the requirements for precise positioning. Overall, the proposed pose forward solution prediction model proves feasible for FSP pose prediction. Compared with the Newton-Raphson method and the traditional BP neural network, this method not only effectively avoids the limitations of the calculation process but also significantly improves computational efficiency and accuracy.

## Conclusions

This paper presents an in-depth investigation into the forward and inverse kinematic solutions of the Flexible Stewart Platform (FSP). In terms of kinematic solving, innovative solutions are proposed from the dimensions of both inverse and forward kinematics, and the effectiveness of these methods is verified through theoretical analysis and simulation experiments. Regarding inverse kinematics, to address the interference problem where the telescopic legs fail to achieve the expected positioning effect due to overcoming the gravity of the moving platform during motion, a novel method utilizing piezoelectric ceramics to calculate the displacement loss is proposed. Simulation results demonstrate that the calculated displacement loss range covered by the piezoelectric ceramics spans from 27.9 nm to 47.4 nm. In terms of forward kinematics, an innovative solution based on an improved BP neural network using the Levenberg-Marquardt (L-M) algorithm is proposed. Simulation results indicate that while maintaining high computational efficiency, the relative error between the predicted pose parameters and the initial pose parameters is controlled within 0.5% across the entire domain, and the absolute error in the heatmap is controlled around 0.02 mm, significantly improving the solution accuracy. By employing the innovative forward kinematic method and the piezoelectric ceramic displacement range compensation technology, this study validates the superior performance of the FSP in the field of high-precision positioning. In the future, this research can be further extended to other industrial and scientific fields requiring precise positioning.
